# Clinician perception of care at the end of life in a quaternary neonatal intensive care unit

**DOI:** 10.3389/fped.2023.1197360

**Published:** 2023-06-13

**Authors:** Lauren Imai, Megan M. Gray, Brennan J. H. Kim, Allison N. J. Lyle, Amber Bock, Elliott Mark Weiss

**Affiliations:** ^1^ Division of Neonatology, Children’s Hospital Colorado, Aurora, CO, United States; ^2^ Department of Pediatrics, University of Washington School of Medicine, Seattle, WA, United States; ^3^ Department of Hospital Medicine, Seattle Children’s Hospital, Seattle, WA, United States; ^4^ Treuman Katz Center for Pediatric Bioethics, Seattle Children’s Research Institute, Seattle, WA, United States

**Keywords:** end of life care, palliative care, symptom management, NICU, neonatal death, quality of death

## Abstract

**Introduction:**

Care for neonates at the end of life (EOL) is often challenging for families and medical teams alike, performed suboptimally, and requires an experienced and compassionate clinician. Much literature exists on adult and pediatric EOL care, but limited studies examine the neonatal process.

**Methods:**

We aimed to describe clinicians' experiences around EOL care in a single quaternary neonatal intensive care unit as we implemented a standard guideline using the Pediatric Intensive Care Unit-Quality of Dying and Death 20 tool.

**Results:**

Surveys were completed by 205 multidisciplinary clinicians over three time periods and included 18 infants at EOL. While most responses were high, a meaningful minority were below goal (<8 on 0–10 scale) for troubling symptom management, conflict between parents and staff, family access to resources, and parent preparation of symptoms. Comparison between Epochs revealed improvement in one symptom management and four communication categories. Satisfaction scores related to education around EOL were better in later Epochs. Neonatal Pain, Agitation, and Sedation Scale scores were low, with few outliers.

**Discussion:**

These findings can guide those aiming to improve processes around neonatal EOL by identifying areas with the greatest challenges (e.g., conflict management) and areas that need further study (e.g., pain management around death).

## Introduction

Infants dying during the neonatal period comprise the largest proportion of childhood deaths and are primarily caused by congenital malformations, chromosomal abnormalities, and prematurity (1). Most of these cases are cared for in the hospital setting and primarily in the neonatal intensive care unit (NICU) (2, 3). End of life (EOL) care is a subset of palliative care focusing on pain and symptom management and family support around the time of death (4). Neonatal EOL care encompasses a respectful, dignified, and family-centered approach that requires many team member activities, resources, and considerations.

To most families, the neonatal dying process and the NICU environment are unfamiliar. The American Academy of Pediatrics (AAP) recommends the inclusion of anticipatory guidance, discovering and incorporating family wishes, and mental health and bereavement services when providing family support at EOL (5). An increasing number of studies have examined parental satisfaction at neonatal EOL, though these share the potential for response bias (6). Studies demonstrate the importance of shared decision-making (7, 8) and the relief of pain and suffering at EOL (7, 9) to improve parental satisfaction. Addressing symptoms and reducing infant suffering may decrease the risk for parental post-traumatic stress symptoms (9).

Evidence supporting optimal pain and symptom management at neonatal EOL is limited. Recent reviews indicate neonatal pain may generally be underestimated due to difficult interpretation of symptoms (10) and off-label pharmaceutical use (11). In a mixed-method study examining the unmet needs of bereaved parents in the NICU, parents report that alleviating infant suffering could be improved (7). Other studies report a lack of pain or symptom documentation, especially after treatment withdrawal (12). In a systematic review examining the family perception of barriers and facilitators of pediatric EOL symptom management, parents believed medication treatments were underutilized, leading to poor pain control at EOL (13).

NICU staff often feel under-supported in providing neonatal EOL care and may benefit from improved education. In a recent survey of U.S. and Canadian neonatologists, neonatal fellow physicians, neonatal nurses, and neonatal nurse practitioners, more than 90% of respondents felt additional education and training in neonatal EOL care would be beneficial (14). In a survey of graduating neonatology fellows, 93% desired education on how to discuss goals of care and family decision-making and yet 41% had no formal training on communicating with families of critically ill patients, especially in the context of religious or spiritual concerns (15). This lack of education is problematic both in terms of under-supporting clinicians and as a barrier to the delivery of optimal care at pediatric and neonatal EOL (13).

Supporting clinicians who provide care during neonatal EOL remains a substantial unmet need. Caring for families during the EOL process is challenging, even in the best circumstances, amongst experienced and compassionate clinicians. The death of a patient in the NICU can cause significant stress on the clinician team, including compassion fatigue (14). In a review of nurses' experience caring for infants at end-of-life, moral distress and feeling of professional inadequacy were considerable challenges identified following patient deaths (16).

Our overall goal is to contribute to knowledge that may improve the dying experience for infants, families, and clinicians. In the current project, we aimed to assess clinician perception of care at the end of life in the setting of the role out of clinical guidelines to support those caring for babies at EOL.

## Methods

We convened a multi-site, multi-disciplinary workgroup of neonatologists, palliative care physicians, nurse practitioners, pharmacists, registered nurses, and social workers to better support EOL practices at our institution. As part of this, we both: (1) crafted clinical guidelines for neonatal EOL and (2) created a method to evaluate neonatal EOL at our quaternary referral NICU.

The group performed a literature review and thorough consensus defined project scope for guideline creation. The guideline included: ethical considerations, preparation for death, symptom management, medication dosing and titration, compassionate extubation guidance, anticipatory guidance for family, steps after death, communication strategies, and clinician support.

To assess clinician perception of EOL, we utilized the Pediatric Intensive Care Unit-Quality of Dying and Death 20 (PICU-QODD-20) survey with minimal modification for the NICU (terminology changed from “child” to “infant”). This tool was developed to assess clinician evaluation of the quality of death and dying in the pediatric intensive care unit and has demonstrated reliability among PICU nurses and physicians (17). Queried components included: pain and symptom management, communication, decisions to withdraw life support, privacy, family, physical, spiritual, and emotional support, fulfilling the parental role, continuity of care, and bereavement.

In addition to the PICU-QODD-20 survey questions, clinicians were asked to rate their satisfaction with current unit guidelines, EOL education level, and clinician support. Respondents used a 5-point likert scale to address satisfaction. Clinicians answered if they reviewed and incorporated new guidelines into practice, if additional resources were required, and inserted free text comments if necessary. We queried demographics, including role, experience in years, age, gender, race, and ethnicity.

We collected data over three time periods. In Epoch 1, we invited respondents to answer questions considering recent neonatal deaths for which they had provided EOL care. During Epoch 2, the development of guidelines, and Epoch 3, the implementation of guidelines, we contacted clinicians after each patient death and asked them to share their experience around that death. Epoch 2 occurred over a 5 month period and Epoch 3 over 6 months.

Care team members for Epoch 2 and 3 were identified via chart review and invited to participate in our survey if they had cared for the infant within 72 h of death. To respect an often stressful or sensitive time, clinician emails were sent 1 week following a patient death. Clinicians were asked to respond within 4 weeks. Target team members included: attending physicians, neonatology fellows, pediatric residents, neonatal nurse practitioners, neonatal physician assistants, and registered nurses. Survey invitations were sent via email and completed on a secure online system.

To better describe the population, we performed a targeted data extraction for each infant death during Epochs 2 and 3. Items included: clinical services consulted, pain management, infant demographics, decision-maker, cause of death, respiratory status prior to death, limited support status, and pain management [including medications given in the last 72 h of life, N-PASS (Neonatal Pain, Agitation, and Sedation Scale) scores within the last 24 h of life, pain interventions and outcomes documented].

Our primary outcome was to describe PICU-QODD-20 scores for clinicians caring for infants at EOL in our NICU. For analyses and interpretation of PICU-QODD responses, we trichotomized 0–10 scale responses into major challenges (0–3), room for improvement (4–7), and meeting goals (8–10). Secondary outcomes included: an exploratory assessment of differences in PICU-QODD-20 responses between Epochs and a description of pain severity in the final 24 h of life.

The study took place at Seattle Children's Hospital, a level IV 32-bed all-out-born NICU, the primary referral center for Washington, Alaska, Idaho, and Montana. The study was approved by the Institutional Review Board at Seattle Children's Research Institute.

## Results

A total of 205 clinicians completed our survey, including 92 out of 250 emailed (37%) in Epoch 1, 54/102 (53%) in Epoch 2, and 59/103 (57%) in Epoch 3. For all three Epochs, most respondents were registered nurses, had spent 0–5 years in their current role, were female, and identified as White and non-Hispanic. Clinician role and demographics did not significantly differ between Epochs and are presented in Table 1.

**Table 1:** Clinicians completing survey.

	Total (*N* = 205)	Epoch 1: baseline (*N* = 92)	Epoch 2: development (*N* = 54)	Epoch 3: guidelines live (*N* = 59)
**Clinician role**				
Attending	36	18	9	9
Fellow	14	4	2	8
Resident	7	0	2	5
Hospitalist	5	1	4	0
NP	24	11	7	6
PA	1	1	0	0
Nurse	118	57	30	31
**Years in current role**				
0–5 years	98	37	29	32
5–9 years	51	26	15	10
10–14 years	25	13	5	7
15 or more years	31	16	5	10
**Gender**				
Female	177	81	45	51
Male	26	10	9	7
Nonbinary	1	0	0	1
**Age**				
18–29 years	58	24	15	19
30–39 years	80	30	26	24
40–49 years	48	25	11	12
50–59 years	10	6	1	3
60–69 years	9	7	1	1
**Race^a^**				
American Indian	3	1	0	2
Asian	24	11	6	7
Black	4	2	1	1
Mixed	1	1	0	0
Native Hawaiian	0	0	0	0
White	173	77	47	49
Unknown	6	1	1	4
**Ethnicity**				
Hispanic	10	6	1	3
Not hispanic	195	86	53	56

*p*-values not significant for all comparisons between three epochs.

^a^ Respondents could choose multiple races.

All eighteen patient deaths in Epoch 2 (*N* = 8) and Epoch 3 (*N* = 10) were included. The infant's median gestational age was in the preterm to late preterm period (36.5 weeks in epoch 2, and 34 weeks in epoch 3). Infants were primarily White, non-Hispanic, and all had parents identified as decision-makers. Most infants died in the NICU and were compassionately extubated prior to death. Some consult services were well utilized (social work, spiritual care), while others were used less frequently (palliative care, ethics) or not at all (pain service). Do-not-resuscitate orders were placed with 3/8 (37%) and 6/10 (60%) patients for Epochs 2 and 3 respectively. All patients received opioids, and most received benzodiazepines, dexmedetomidine, or other sedatives within 72 h of death. Infant clinical and demographic information did not significantly differ between Epochs (Table 2).

**Table 2:** Infant clinical and demographic information.

	Epoch 2 (*N* = 8)	Epoch 3 (*N* = 10)
Completed gestional age in weeks at birth (Median, interquartile range)	36.5 (34, 39)	34 (29,37)
**Sex assigned at birth**		
Male	4	5
Female	4	5
**Race**		
American Indian	1	0
Asian	1	0
Black	1	1
Native Hawaiian	0	0
White	5	5
Unknown^a^	0	4
**Ethnicity**		
Hispanic	0	1
Not hispanic	8	6
Unknown	0	3
**Decision-makers**		
Parents	8	10
**Location of death**		
NICU	5	10
Interventional Cardiology	1	0
NICU team care off unit^b^	2	0
Intubated at death	1	5
Compassionate extubation	6	5
Immediate	6	4
Terminal wean	0	1
**Consults during NICU stay**		
Social work	7	9
Spiritual	6	7
Child life	4	3
Palliative care	4	1
Ethics	0	1
Pain	0	0
DNR placed	3	6
**DNR details**		
Limited non-invasive respiratory support	3	3
Limited invasive respiratory support	3	3
Limited cardiac support	3	6
**Medications within 72 h of death**		
Opioids	8	10
Benzodiazepines	6	7
Dexmedetomidine	6	6
Other sedatives	6	8

*p*-values not significant for all comparisons between Epochs.

^a^ Race data extracted from medical record. For these 4: two “patient refused” one listed as “not inputted” and one had no data.

^b^ NICU team care off unit: outside of NICU physical space but under care of NICU team such as on rooftop garden for a planned compassionate extubation.

**Figure 1:**
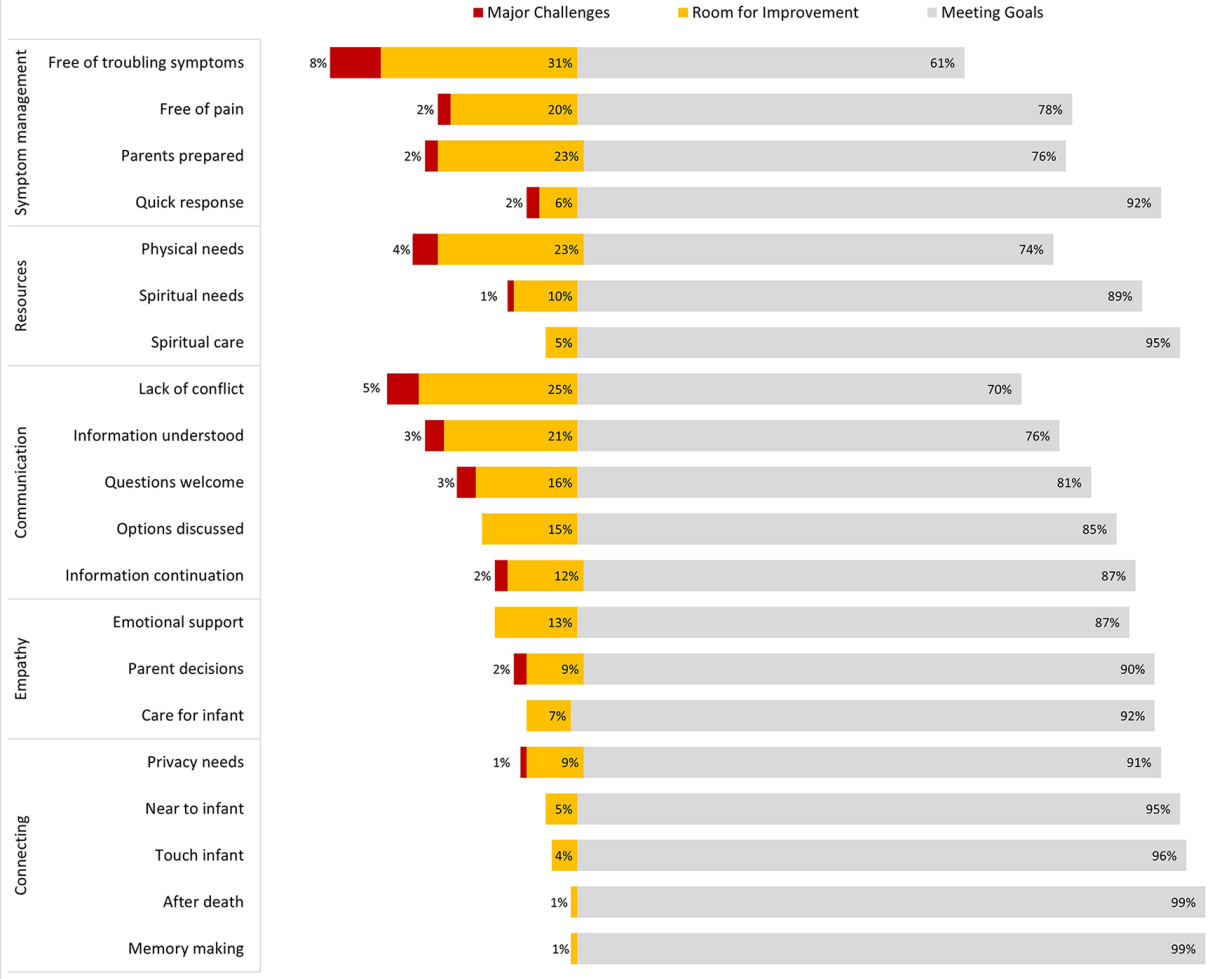
PICU-QQDD trichotomized responses for all questions from all epochs. Responses for all *N* = 205 respondents over three Epochs, 0–10 scale trichotomized into: major challenges (0–3) room for improvement (4–7) and meeting goals (8–10).

Most responses to PICU-QODD-20 were “meeting goal” (score of 8–10 on 0–10 scale) for each question; however, a significant minority selected responses categorized as “major challenges” (0–3) or “room for improvement” (4–7). Questions demonstrating major challenges included troubling symptom management (8% major challenges, and 31% room for improvement), a conflict between parents and staff (5% major challenges, 25% room for improvement), family access to resources (4% major challenges, 23% room for improvement), and parent preparation of symptoms (2% major challenges, 23% room for improvement). For all Epochs, the highest scoring questions were within the “connecting” category: 96% of staff surveyed reported being near or at goal in providing the opportunity for families to connect with their infant at EOL. Survey responses for the PICU-QODD-20 are presented in Figure 1. PICU-QODD-20 categories and questions are presented in Table 3.

**Table 3:** PICU-QODD-20 questions converted to NICU context by category.

Category	Item	Full text of item
Symptom management	Free of troubling symptoms	The infant was free of other troubling symptoms
	Free of pain	The infant was free of pain
	Parents prepared	Staff prepared parents for what might happen to their infant
	Quick response	Staff responded quickly to parents’ concerns about their infant's symptoms
Resources	Physical needs	Parents found it easy to meet their basic physical needs (accessible bathroom, showers, affordable meals, places to stay, parking, etc.)
	Spiritual needs	Staff discovered and respected the family's spiritual and/or religious needs
	Spiritual care	Hospital spiritual care was available
Communication	Lack of conflict	There were no conflicts between parents and the clinical staff about the best way to care for the infant
	Information understood	Staff gave parents information about their infant in a way that they could understand
	Questions welcome	Staff created an atmosphere in which parents felt comfortable asking questions about their infant
	Options discussed	Staff offered parents opportunities to discuss options about their infant's care with the healthcare team
	Information continuation	Nurses and doctors did a good job of passing information about the infant onto the next shift or rotation
Empathy	Emotional support	Staff supported the parents emotionally
	Parent decisions	Staff discovered and respected parents’ wishes and decisions
	Care for infant	Staff demonstrated that they cared about the infant as an individual
Connecting	Privacy needs	Staff provided parents with privacy with their infant near the end of their infant's life
	Near to infant	Staff provided parents with opportunities to be near their infant
	Touch infant	Staff helped parents find ways to touch, hold, and/or connect with their infant
	After death	Once the infant died, parents were allowed to stay with them for as long as they wanted
	Memory making	Staff helped parents create memories (such as handprints, lockets of hair, photographs) of their infant

Planned secondary analysis revealed differences in scores by Epoch for more than half of the questions (Table 4). Because we found significant differences, we then performed *post-hoc* pairwise comparison testing. There were no differences between Epochs 2 and 3. Comparisons between Epochs 1–2 and Epochs 1–3 revealed improvement in one symptom management question (parents prepared), one resource question (spiritual needs), and four communication questions (lack of conflict, information understood, questions welcome, and options discussed). *Post-hoc* pairwise comparisons are presented in Table 5.

**Table 4:** PICU-QODD-20 scores by Epoch.

Category	Item	Total	Epoch 1	Epoch 2	Epoch 3	*p*-value
		Median (IQR)	Median (IQR)	Median (IQR)	Median (IQR)	
Symptom management	Free of troubling symptoms	8 (6, 9)	8 (6, 9)	8 (7, 9)	8 (6, 9)	0.28
	Free of pain	9 (8, 9)	8 (7, 9)	9 (8, 9)	9 (8, 10)	0.19
	Parents prepared	9 (8, 10)	8 (7, 9)	9 (8, 10)	9 (8, 10)	0.0001
	Quick response	9 (8, 10)	9 (8, 10)	9 (9, 10)	10 (8, 10)	0.01
Resources	Physical needs	9 (7, 10)	8 (7, 9)	9 (8, 10)	9 (8, 10)	0.007
	Spiritual needs	9 (8, 10)	9 (8, 10)	10 (9, 10)	10 (9, 10)	0.001
	Spiritual care	9 (9, 10)	9 (8, 10)	10 (9, 10)	10 (9, 10)	0.01
Communication	Lack of conflict	9 (7, 10)	8 (7, 9)	10 (9, 10)	9 (6, 10)	0.0001
	Information understood	9 (8, 10)	8 (7, 9)	9 (8, 10)	9 (8, 10)	0.0001
	Questions welcome	9 (8, 10)	8 (7, 9)	9 (8, 10)	9 (8, 10)	0.0001
	Options discussed	9 (8, 10)	9 (8, 9)	10 (9, 10)	10 (9, 10)	0.0001
	Information continuation	9 (8, 10)	9 (8, 10)	10 (9, 10)	9.5 (8, 10)	0.007
Empathy	Emotional support	9 (8, 10)	9 (8, 10)	10 (8, 10)	9.5 (8, 10)	0.04
	Parent decisions	9 (8, 10)	9 (8, 10)	10 (9, 10)	10 (8, 10)	0.004
	Care for infant	10 (9, 10)	9 (9, 10)	10 (9, 10)	9.5 (8, 10)	0.14
Connecting	Privacy needs	9 (9, 10)	9 (8, 10)	10 (9, 10)	10 (9, 10)	0.002
	Near to infant	10 (9, 10)	10 (9, 10)	10 (9, 10)	10 (9, 10)	0.17
	Touch infant	10 (9, 10)	10 (9, 10)	10 (9, 10)	10 (9, 10)	0.66
	After death	10 (10, 10)	10 (9, 10)	10 (10, 10)	10 (10, 10)	0.05
	Memory making	10 (9, 10)	10 (9, 10)	10 (10, 10)	10 (9, 10)	0.01

Kruskal Wallis *p*-values.

**Table 5:** PICU-QODD-20 scores by epoch, *post-hoc* pairwise comparisons.

Category	Item	Epoch 1 vs. 2	Epoch 1 vs. 3	Epoch 2 vs. 3
Symptom management	Free of pain	0.60	0.68	0.73
	Free of troubling symptoms	0.58	0.49	0.97
	Parents prepared	0.03	0.0001	0.17
	Quick response	0.29	0.15	0.16
Resources	Physical needs	0.16	0.24	0.81
	Spiritual needs	0.04	0.03	0.76
	Spiritual care	0.21	0.13	1
Communication	Lack of conflict	0.0001	0.0001	0.09
	Information understood	0.001	0.0001	0.42
	Questions welcome	0.002	0.0001	0.26
	Options discussed	0.01	0.004	0.87
	Information continuation	0.02	0.08	0.63
Empathy	Emotional support	0.09	0.05	0.53
	Parent decisions	0.03	0.17	0.73
	Care for infant	0.07	0.79	0.28
Connecting	Privacy needs	0.09	0.16	0.33
	Near to infant	0.61	0.44	1
	Touch infant	0.13	0.75	0.10
	After death	0.15	0.51	0.50
	Memory making	0.02	0.44	0.24

Presented values are *p*-values for pair-wise comparisons.

Analysis of additional questions on clinician surveys revealed interpreter availability scored relatively poorly, with 6% major challenges and 28% room for improvement. Knowledge of and use of the guideline post-implementation was also limited, with slightly more than half (30/58) of clinicians reporting they had reviewed the EOL guideline in Epoch 3. Women were much more likely to report that they had reviewed guidelines than men.

**Figure 2:**
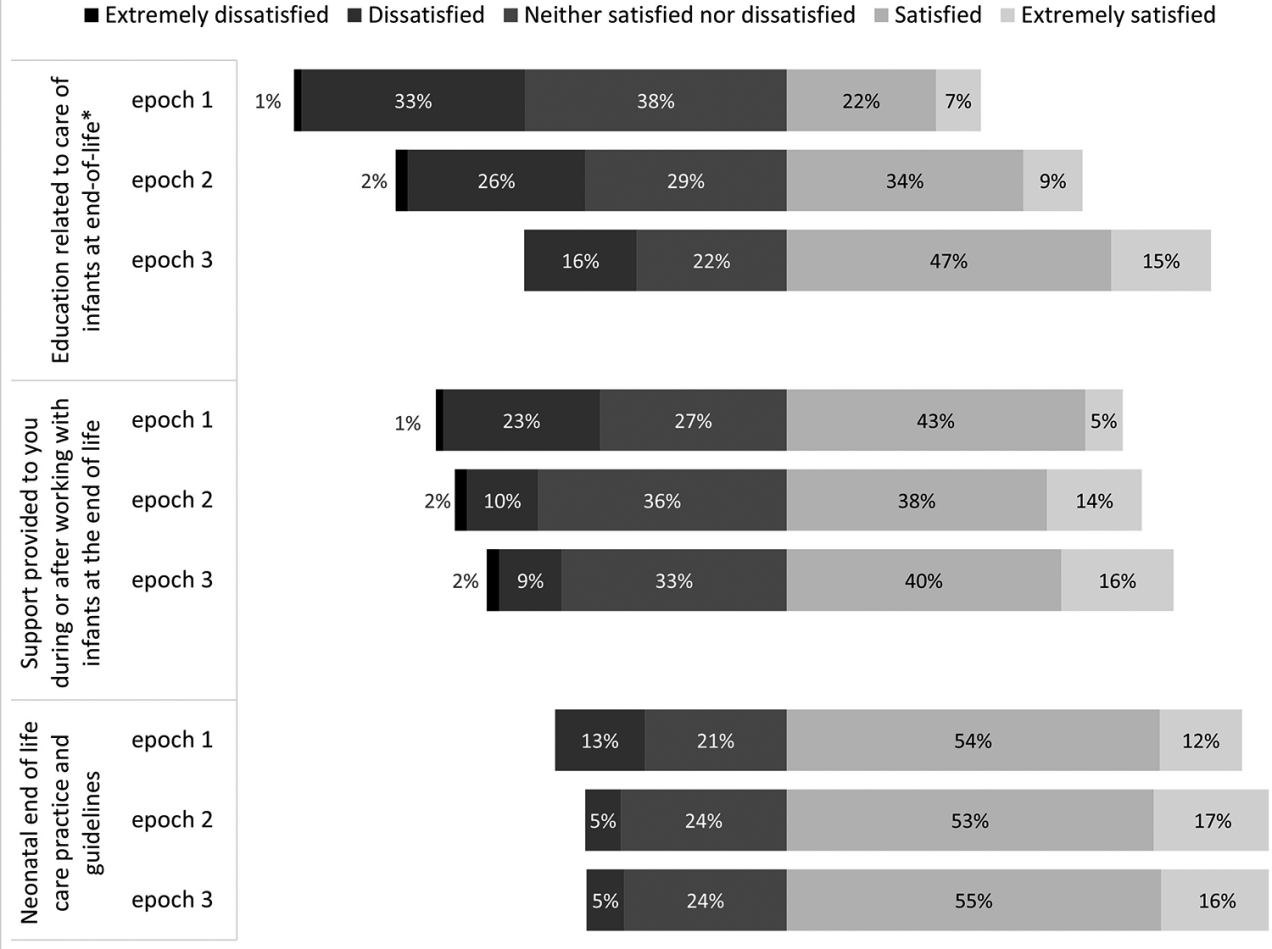
Satisfaction scores by epoch. Responses by Epoch to three 5-point Likert scale questions.

Satisfaction with education related to EOL care significantly improved over the three Epochs, while satisfaction with staff support and EOL guidelines did not change significantly around the epochs. Satisfaction scores were compared using Fisher's exact test and are presented in Figure 2.

**Figure 3:**
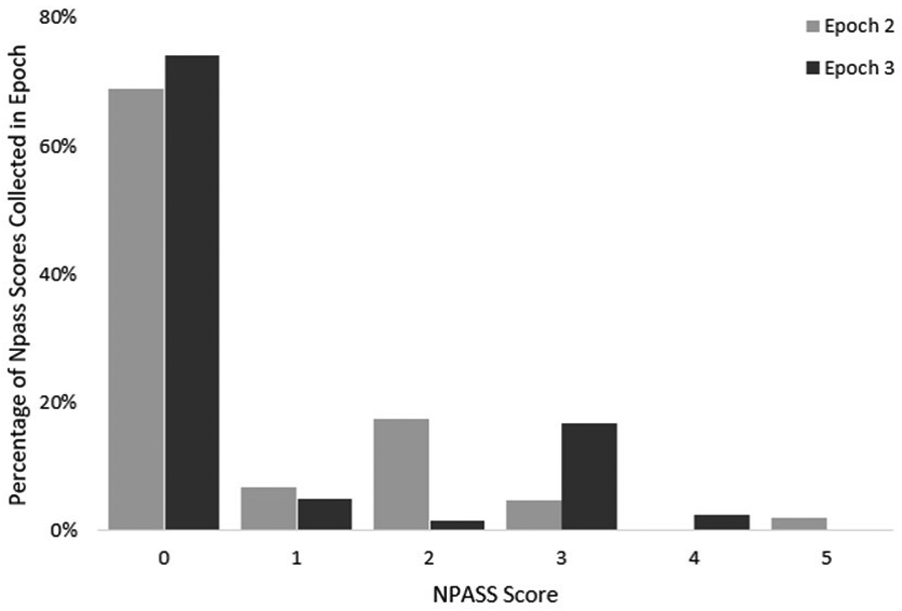
NPASS scores within 24 h of death.

NPASS scores during the 24 h prior to death were reviewed. A total of 223 NPASS scores were included. Scores were documented at variable intervals and variable relation to pharmacological and non-pharmacological interventions. At EOL, many infants had low pain scores (0–3), though some outliers were noted. NPASS scores did not differ between Epochs 2 and 3. NPASS scores are presented in Figure 3.

Three of the patient deaths occurred rapidly or unexpectedly. In Epoch 1, a patient's death occurred unexpectedly during a procedure off the unit. In Epoch 2, there were two outliers, with one infant experiencing rapid decompensation and redirection of care decision-making by phone with the parents and a second infant who arrived at the unit actively receiving cardiopulmonary resuscitation and subsequently died.

## Discussion

The responses to this survey study revealed several findings that may be important to others aiming to improve care around neonatal EOL that warrant further discussion. First, PICU-QODD-20 responses revealed areas of improvement related to EOL care within categories of symptom management, resource availability, and communication. Within symptom management, addressing non-pain symptoms and adequately preparing parents for death frequently scored poorly. Within resource availability, family access to basic resources scored poorly. Within communication, a conflict between staff and parents, and providing information to parents in understandable ways also scored poorly. Second, comparisons by Epoch showed improvement comparing Epoch 1 to Epochs 2 and 3, most notably within the communication category. Third, we will consider findings related to NPASS scores and medication administration.

Similar to older children at EOL, neonates may exhibit agitation, restlessness, dyspnea, excessive secretions, constipation, neuroirritability, or seizures at EOL (18). Managing such symptoms at EOL frequently scored poorly on our surveys. Identifying EOL symptoms in neonates presents challenges for the clinician, as symptoms may be confused with hunger or the need for parental support and holding. Non-pharmacological symptom management (e.g., bundling) at EOL frequently lacks documentation (12), leading to difficulties in understanding how to manage such symptoms better. In our chart audit, N-PASS scores were inconsistently repeated after medication administration. Missing data led to challenges in determining how to improve this issue in our unit. Future work addressing troubling symptoms at EOL may include improvement in identification and adequate documentation of patient response.

Adequately preparing parents for infant death also frequently scored poorly on our surveys. The importance of this step often falls upon the bedside nursing staff. The National Association of Neonatal Nurses (NANN) position statement on palliative and EOL care for neonates lays out the need for family preparation to include anticipatory guidance for the timeline of death and expected symptoms such as dyspnea (19). In our study, some infants experienced an abrupt redirection of care, leading to limited time during the move from life-sustaining measures to withdrawal. Completing tasks during and around neonatal EOL may limit the ability of clinicians, in particular bedside nurses, to provide emotional support to parents. All but two sets of parents in our cohort were able to be with their child at EOL. Those families that were not present, arrived shortly after. In these cases, adequate preparation is unfortunately incredibly challenging. Parent information needs around neonatal EOL are variable and dynamic (20). Too much, too little, or the wrong type of information may overwhelm parents and engender fear and distress (21). Clinicians may benefit from additional educational, emotional, and logistical support around preparing parents for neonatal EOL.

A challenging aspect of EOL care identified by our survey included conflict between parents and clinical staff about the best way to care for the infant. The core of such issues may stem from differing values between clinicians and family members. Most parents want decision-making control related to neonatal EOL care (22), but how they operationalize this can vary greatly. The deeply personal and varied parental beliefs, values, and preferences around neonatal EOL should be considered in training for those providing neonatal EOL care (23, 24). For our families in this study, decision-making related to withdrawal looked different with each case. Some chose the location of where to withdraw (a garden, or laying in a bed), some were active participants in pain relief planning, some chose religious ceremony or memory-making with professional photography. The clinicians on the unit worked to discover parental wishes and respect those choices, but some conflicts did arise. For one infant, rapid decompensation led to the a death in which family could not be present. As described in free-text comments, the physician had the mother on speaker phone as the decision was made to halt life-saving measures. Many clinicians reported feeling distressed when this occurred, and that the medical team should've initiated communication with family sooner.

In our unit, consulting services such as ethics and palliative care were readily available to assist clinicians and families with conflict resolution. One infant during our study period received an ethics consult for perceived futility of care, and five infants (28%) had palliative care consultations, similar to usage in other published cohorts (25). Palliative care consultation in pediatric EOL has been associated with shorter length of stay, less use of invasive interventions, and death outside of the ICU (26). In the NICU, palliative care consultation has been associated with increased care redirection and palliative medication use in the last 48 h of life (27). Although the AAP recommends palliative care consultation for any pediatric patient experiencing a life-limiting illness (5), there is variability among U.S. NICUs (14). Whether all neonates at EOL can, or should, receive palliative care consultations is an open question.

Responses frequently indicated major challenges related to physical resources for family, such as access to bathrooms, showers, meals, accomodations, and parking. In our unit, all rooms were newly built single-patient rooms, and parents were allowed to stay at bedside all night and had access to a private shower and bathroom. Breastfeeding mothers were provided free meals delivered to their rooms to support self-care. The study period overlapped with the COVID-19 pandemic, in which visitation policies were variably restricted and off-unit support, such as family resource centers were closed. In addition, because our NICU receives referrals from three other states, it is possible some families had fewer resources available, simply because they were further from home. Our study did not follow family's home location, but these challenges may have contributed to lower scores. In one survey free text response, a clinician noted difficulty with transportation for the father. Provision of basic needs, such as transportation, should ideally be offered to support family-centered care for all NICU families (28). Within the context of neonatal EOL, these are critical to enabling parents to be with their infant near the time of death. In other free text responses, free family housing was denied and parents were unable to purchase food overnight when the cafeteria closed. Our findings suggest that even well-funded, quaternary academic research centers with multiple levels of support for families sometimes fail to meet the physical needs of families at EOL.

Items showing improvement from Epoch 1 to Epoch 2 and 3 included parent preparation as part of symptom management, conflict between staff and family, providing understandable information, questions welcomed, and options discussed. It is possible clinicians felt more comfortable preparing the family for infant death following the education provided in EOL guidelines, as satisfaction regarding education significantly improved over the epochs as well. No items worsened over the epochs.

Infant N-PASS scores 24 h before death were generally low (0–3) but with some high (unit goal of N-PASS less than or equal to 3). One patient experienced high scores that persisted for hours at EOL, despite pharmaceutical interventions. Though N-PASS was not created for EOL care, it is a reasonable tool to use during the acute pain process, and the NANN palliative care position statement supports its use (20). The N-PASS is validated for use in mechanically ventilated infants and a wide range of ages, including down to 23 weeks of gestation (29). Limited prior work has reported actual pain scores at neonatal EOL for comparison to our results. One study describes the lack of consistent pain score documentation at EOL as a barrier to analyzing symptom management for neonates (19). Future work must endeavor to link infant pain scores, medication and non-pharmacological management, clinician experience, and parent experience around neonatal EOL.

## Limitations

Our study has multiple limitations. We only included the views of clinicians, not parents. Parental surveys were not possible logistically, and we noted a low response rate and response bias in other studies related to EOL ICU care (6). Future work must include parental experiences. Participants were recruited from a single center, which may limit generalizability. Methodologically, the difference between Epoch 1 asking about “recent deaths” and Epochs 2 and 3 asking about a specific recent death may have contributed to variation in responses in unknown ways. Clinicians may have contributed to the dataset more than once if they took care of multiple infants at EOL; our methodologies did not enable us to link these responses or to evaluate for potential change over time for a given clinician. Finally, Epochs 2 and 3 may not have been as distinct as ideal, as many clinicians were involved in or aware of the development of guidelines during Epoch 2: some respondents during Epoch 2 may have been influenced by the contents of the guidelines. Alternatively, some respondents during Epoch 3 may have been unaware of the guidelines.

## Conclusion

In this survey study of clinicians providing neonatal EOL care, we identified key items for future exploration and intervention development, notably troubling symptom management, a conflict between parents and staff, family access to resources, and parent preparation of symptoms. Our findings may inform future work endeavoring to improve the care of neonates at EOL and their families.

## Data Availability

The original contributions presented in the study are included in the article, further inquiries can be directed to the corresponding author.

## References

[1] Centers for Disease Control and Prevention. Infant mortality. Available at: https://www.cdc.gov/reproductivehealth/maternalinfanthealth/infantmortality.htm (Accessed March 7, 2023).

[2] Brandon D Docherty SL Thorpe J. Infant and child deaths in acute care settings: implications for palliative care. J Palliat Med. (2007) 10(4):910–8. 10.1089/jpm.2006.0236 17803413

[3] NHPCO facts and figures: pediatric palliative and hospice care in America. National Hospice and Palliative Care Organization (2014).

[4] National Coalition for Hospice and Palliative Care. Clinical practice guidelines for quality palliative care, 4th edition. Available at: https://www.nationalcoalitionhpc.org/wp-content/uploads/2020/07/NCHPC-NCPGuidelines_4thED_web_FINAL.pdf (Accessed November 20, 2022).

[5] Section on Hospice and Palliative Medicine and Committee on Hospital Care. Pediatric palliative care and hospice care commitments, guidelines, and recommendations. Pediatrics. (2013) 132(5):966–72. 10.1542/peds.2013-2731 28448256

[6] Kross EK Engelberg RA Shannon SE Curtis JR. Potential for response bias in family surveys about end-of-life care in the ICU. Chest. (2009) 136(6):1496–502. 10.1378/chest.09-0589 19617402 PMC2789922

[7] Baughcum AE Fortney CA Winning AM Dunnells ZDO Humphrey LM Gerhardt CA. Healthcare satisfaction and unmet needs among bereaved parents in the NICU. Adv Neonatal Care. (2020) 20(2):118–26. 10.1097/ANC.0000000000000677 31569093

[8] Brosig CL Pierucci RL Kupst MJ Leuthner SR. Infant end-of-life care: the parents’ perspective. J Perinatol. (2007) 27(8):510–6. 10.1038/sj.jp.7211755 17443196

[9] Clark OE Fortney CA Dunnells ZDO Gerhardt CA Baughcum AE. Parent perceptions of infant symptoms and suffering and associations with distress among bereaved parents in the NICU. J Pain Symptom Manage. (2021) 62(3):e20–7. 10.1016/j.jpainsymman.2021.02.015 33631329

[10] Haug S Dye A Durrani S. End-of-life care for neonates: assessing and addressing pain and distressing symptoms. Front Pediatr. (2020) 8:574180. 10.3389/fped.2020.574180 33072678 PMC7542096

[11] Garten L Bührer C. Pain and distress management in palliative neonatal care. Semin Fetal Neonatal Med. (2019) 24(4):101008. 10.1016/j.siny.2019.04.008 31056417

[12] Fortney CA Steward DK. Medical record documentation and symptom management at the end of life in the NICU. Adv Neonatal Care. (2015) 15(1):48–55. 10.1097/ANC.0000000000000132 25313801 PMC4310764

[13] Greenfield K Holley S Schoth DE Harrop E Howard RF Bayliss J A mixed-methods systematic review and meta-analysis of barriers and facilitators to paediatric symptom management at end of life. Palliat Med. (2020) 34(6):689–707. 10.1177/0269216320907065 32228216 PMC7521017

[14] Haug S Farooqi S Wilson CG Hopper A Oei G Carter B. Survey on neonatal end-of-life comfort care guidelines across America. J Pain Symptom Manage. (2018) 55(3):979–984.e2. 10.1016/j.jpainsymman.2017.10.023 29129740

[15] Boss RD Hutton N Donohue PK Arnold RM. Neonatologist training to guide family decision making for critically ill infants. Arch Pediatr Adolesc Med. (2009) 163(9):783–8. 10.1001/archpediatrics.2009.155 19736330 PMC2843907

[16] Gibson K Hofmeyer A Warland J. Nurses providing end-of-life care for infants and their families in the NICU: a review of the literature. Adv Neonatal Care. (2018) 18(6):471–9. 10.1097/ANC.0000000000000533 30507828

[17] Sellers DE Dawson R Cohen-Bearak A Solomond MZ Truog RD. Measuring the quality of dying and death in the pediatric intensive care setting: the clinician PICU-QODD. J Pain Symptom Manage. (2015) 49(1):66–78. 10.1016/j.jpainsymman.2014.05.004 24878067 PMC4247362

[18] Carter BS Jones PM. Evidence-based comfort care for neonates towards the end of life. Semin Fetal Neonatal Med. (2013) 18(2):88–92. 10.1016/j.siny.2012.10.012 23182618

[19] http://nann.org/uploads/About/PositionPDFS/1.4.5_Palliative%20and%20End%20of%20Life%20Care%20for%20Newborns%20and%20Infants.pdf.

[20] Pirrello J Sorin G Dahan S Michel F Dany L Tosello B. Analysis of communication and logistic processes in neonatal intensive care unit. BMC Pediatr. (2022) 22(1):137. 10.1186/s12887-022-03209-1 35291967 PMC8922841

[21] Xafis V Wilkinson D Sullivan J. What information do parents need when facing end-of-life decisions for their child? A meta-synthesis of parental feedback. BMC Palliat Care. (2015) 14:19. 10.1186/s12904-015-0024-0 25924893 PMC4424961

[22] Weiss EM Xie D Cook N Coughlin K Joffe S. Characteristics associated with preferences for parent-centered decision making in neonatal intensive care. JAMA Pediatr. (2018) 172(5):461–8. 10.1001/jamapediatrics.2017.5776 29554176 PMC5875325

[23] Fortney CA. Palliative and end-of-life care for infants and their families in the NICU: building a program of research. J Pediatr Nurs. (2019) 49:104–5. 10.1016/j.pedn.2019.09.019 31668673 PMC6942219

[24] Beltran SJ Hamel MN. Caring for dying infants: a systematic review of healthcare providers’ perspectives of neonatal palliative care. Am J Hosp Palliat Care. (2021) 38(8):1013–27. 10.1177/1049909120965949 33054317

[25] McLaughlin SN Song MK Hertzberg V Piazza AJ. Use of palliative care consultation services for infants with life-threatening conditions in a metropolitan hospital. Adv Neonatal Care. (2020) 20(2):136–41. 10.1097/ANC.0000000000000698 32224820

[26] Keele L Keenan HT Sheetz J Bratton SL. Differences in characteristics of dying children who receive and do not receive palliative care. Pediatrics. (2013) 132(1):72–8. 10.1542/peds.2013-0470 23753086

[27] Samsel C Lechner BE. End-of-life care in a regional level IV neonatal intensive care unit after implementation of a palliative care initiative. J Perinatol. (2015) 35(3):223–8. 10.1038/jp.2014.189 25341197

[28] Craig JW Glick C Phillips R Hall SL Smith J Browne J. Recommendations for involving the family in developmental care of the NICU baby. J Perinatol. (2015) 35(Suppl 1):S5–8. 10.1038/jp.2015.142 26597804 PMC4660048

[29] Hummel P Puchalski M Creech SD Weiss MG. Clinical reliability and validity of the N-PASS: neonatal pain, agitation and sedation scale with prolonged pain. J Perinatol. (2008) 28(1):55–60. 10.1038/sj.jp.7211861 18165830

[30] Harris PA Taylor R Minor BL Elliott V Fernandez M O'neal L The REDCap consortium: building an international community of software platform partners. J Biomed Inform. (2019) 95:103208. 10.1016/j.jbi.2019.103208 31078660 PMC7254481

[31] Harris PA Taylor R Thielke R Payne J Gonzalez N Conde JG. Research electronic data capture (REDCap)–a metadata-driven methodology and workflow process for providing translational research informatics support. J Biomed Inform. (2009) 42(2):377–81. 10.1016/j.jbi.2008.08.010 18929686 PMC2700030

